# Evaluation of UV Curing Properties of Mixture Systems with Differently Sized Monomers

**DOI:** 10.3390/ma11040509

**Published:** 2018-03-28

**Authors:** Ji-Won Park, Gyu-Seong Shim, Jong-Gyu Lee, Seong-Wook Jang, Hyun-Joong Kim, Jin-Nyung Choi

**Affiliations:** 1 Lab of Adhesion and Bio-Composite, Program in Environmental Materials Science, Department of forest science, Seoul National University, Seoul 08826, Korea; roorouny@gmail.com (J.-W.P.); sks6567@snu.ac.kr (G.-S.S.); spdh123@snu.ac.kr (J.-G.L.); jangsw0202@snu.ac.kr (S.-W.J.); 2 Research Institute of Agriculture and Life Sciences, Seoul National University, Seoul 08826, Korea; 3 Nano Polychem, Hannam University, Ojeong-dong, Daedeok-gu, Daejeon 34430, Korea; nanotsr@nnpc.co.kr

**Keywords:** mixture system of acrylate, photo polymerization, UV-curing, shrinkage, nano-porous effect, synergy effect

## Abstract

Ultraviolet (UV) curing is a photopolymerization technique resulting in a three-dimensional polymer network from monomers and oligomers after exposure to UV light, which is often used for fusion industry. However, shrinkage is an issue that needs to be resolved. Studies of single substances have been extensively conducted, but studies of mixture systems have not sufficiently been undertaken. In this study, we evaluate the shrinkage phenomenon by studying a monomer/monomer binary system and monomer/macromer composite systems. Shrinkage tends to increase when compounds varying in size are used. Similar to the shrinkage phenomenon, the curing rate is also relatively higher in such systems. These synergistic effects are evaluated to be due to the nano-porous effect, and vary with the composition ratio and material structure.

## 1. Introduction

Ultraviolet (UV) curing systems have recently been utilized in various industries. They are used not only for general industries such as surface coatings, manufacturing functional film, or exterior coatings of vehicles, but also for forming 3D dimensional structure and pattern masks in semiconductor processes [1,2,3].

When a photoinitiator contained in a UV curing system is exposed to UV light, a photopolymerization reaction is initiated; the monomers and oligomers, which are the main components of the polymer resin, instantaneously form a three-dimensional (3D) network polymer. This type of UV curing technology has many advantages. As it is capable of rapid curing and constitutes a process that is low in volatile organic compounds (VOCs), it has been a key coating technology. However, while this UV curing technology has various advantages, some issues need to be addressed. Among them, the shrinkage phenomenon is considered to be a problem, since it can affect the shape and durability of materials [4,5,6].

During UV curing, shrinkage is caused by overlapping spaces that single molecules occupy, i.e., the sum of free volumes of unit molecules is larger than the free volume of a polymer formed from those unit molecules. This difference in space allows shrinkage, which causes various problems during the curing process. First, it reduces adhesion to the surface to be coated. In addition, it is aesthetically problematic as it causes the surface to become uneven [7,8]. This shrinkage of the UV curing system does not cause problems only on the surface. Due to the shrinkage phenomenon, the UV curable material generates unnecessary internal stress [9,10]. This internal stress acts as a major cause of the long-term shape change of the material. For example, in the dental field, internal stresses of the resin cause problems such as interface failure between the resin and the pubis and secondary infection [11,12]. In the case of pressure sensitive adhesives using UV curing process, product defects occur due to thickness variations of the film [13]. Recently, non-uniformity of samples produced through UV 3D printing process has been reported [14,15].

The shrinkage (contraction) phenomenon in systems with one type of molecules has previously been elucidated. Shrinkage is generally known to be inversely proportional to the molecular weights of the unit molecules and proportional to the number of functional groups. Furthermore, it also varies with molecular mobility and the environmental conditions of the curing system. However, UV-curable materials can vary in composition depending on the application field. UV curing systems consist of multiple components including monomers, oligomers, photoinitiators, fillers, and a wide range of additives [8,16]. The shrinkage properties presented in the above assessment are limited to the raw material. In a multi-component system, it is difficult to directly observe trends in changes in the physical properties owing to the presence of many variables. Therefore, it is important to analyze the results depending on multiple parameters by simplifying each system as much as possible.

The analysis of UV curing behavior has been described in various ways. It is important to evaluate the instantaneous rate of change since UV curing is very rapid. Analysis of the reaction heat through Photo-Differential Scanning Calorimetry (DSC) has been mainly used to evaluate the reaction rate. Photo-DSC analysis shows that the reaction rate varies depending on the structure of the monomer and the type of the functional group [4,17,18]. However, since the energy difference due to the conformation of the polymer is reflected, the accurate conversion rate cannot be calculated. Real-time Real-time Fourier Transform Infrared Spectroscopy (FT-IR) has been used to supplement this evaluation. The evaluation of UV curing behavior using FT-IR is directly comparable to the change in C = C, allowing intuitive interpretation [19,20]. These two methods are known to have similar tendencies but to have large differences in results. Therefore, its application is limited depending on the composition of the UV curing system [21,22].

Shrinkage evaluation in UV curing systems has been used to verify the stability of materials. A linometer was introduced in the dental field and was used to evaluate the curing properties of dental resins [23]. In recent years, evaluation of UV curing kinetics through evaluation of shrinkage rate has been introduced. Shrinkage means total conversion of UV-curable resin, so the conversion rate can be intuitively explained. It is known that photo-DSC can be used in parallel with complementary use. However, these studies are limited to mono-material systems. Therefore, there is a continuing need for interpretation of multicomponent systems [8,24].

In recent years, composite materials of acrylates with fillers have been increasingly used to improve the strength and adhesion of coating films as well as inhibit shrinkage phenomena. Fillers exist in a variety of systems such as organic, biological, biomimetic, and polymeric materials. In polymer systems, fillers not only reduce the material costs, but may also improve the mechanical and dynamic properties of the final compounds. A typical example is the introduction of silica particles into a coating material to improve its surface hardness. Silica and other filler types exhibit weak polymer–filler interactions and are extensively used if a high degree of reinforcement is not essential. A silica filler has several advantages, such as an increase in curing stability and improvement of post-curing mechanical properties. However, it may also negatively affect the curing properties and introduce the problem of remnant uncured materials depending on the exact type of filler used [25,26,27].

An example application of composite materials is the common use of polymer matrices with incorporated metal oxides and silica nanoparticles to replace amalgam in dental fillings. Dental filling matrices are typically composed of acrylate- or methacrylate-based monomers that can be photopolymerized, chemically polymerized, or dual-cured, and strong adhesion accompanied by minimal shrinkage is essential for such dental applications [14,28,29].

Considering the above, the elucidation of shrinkage phenomenon in complex composite materials containing silica fillers is needed. In this study, therefore, a monomer/monomer binary blended system and monomer/filler (macromer) composite system were used to analyze the shrinkage phenomena that occur when either a single material is used or when materials are mixed.

## 2. Experimental

### 2.1. Blended System Containing Multifunctional Acrylates

**Figure 1:**
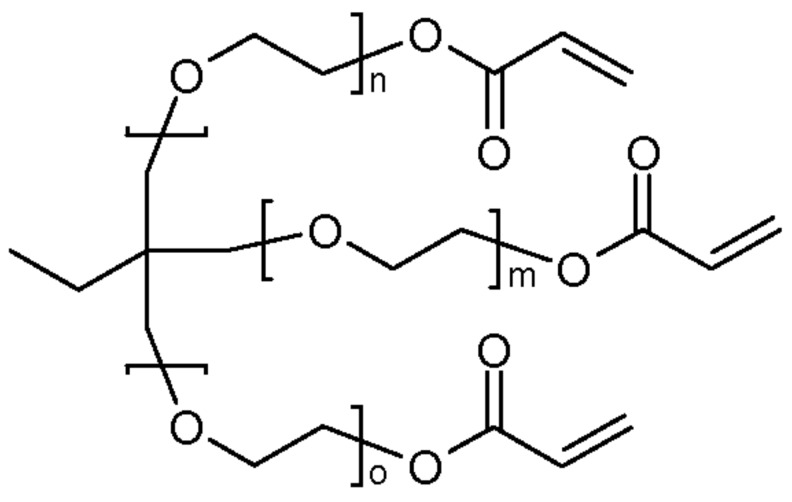
Structure of trimethylolpropane triacrylate (TMPTA) and ethoxylated TMPTA (EO6TMPTA): (**a**) TMPTA: n + m + o = 0; and (**b**) EO6TMPTA: n + m + o = 6.

Here, we used trimethylolpropane triacrylate (TMPTA, Sartomer, Rieux, France) and ethoxylated (6) trimethylolpropane triacrylate (EO6TMPTA, Sartomer, Rieux, France), commonly used as multi-functional acrylates, to evaluate blended binary systems (Figure 1). The core structures of the two materials are the same, although the chain length of EO6TMPTA differs from that of TMPTA. These materials were selected to evaluate UV curing changes when two materials of different molecular sizes are mixed. Five specimens were prepared from TMPTA and EO6TMPTA with weight percentage ratios of 100:0, 75:25, 50:50, 25:75, and 0:100, for which photo-DSC and shrinkage evaluations were conducted. Hydroxydimethyl acetophenone (Micure HP-8, Miwon Specialty Chemical, Anyang-si, Korea) was used as the photoinitiator. The main absorption ranges were 265–280 nm and 320–335 nm.

### 2.2. Filler-Containing Acrylate Systems

A method to produce nano-silica particles from tetraethoxysilane (TEOS) has previously been proposed using the sol-gel method [30], and the synthesis of coating materials with nano-silica has recently found extensive use [31,32]. Nonetheless, to effectively utilize nano-silica particles, a surface treatment technique is required that improves their dispersibility.

**Figure 2:**
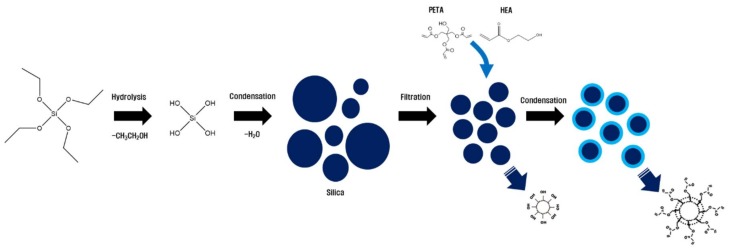
Preparation of the nano-silica core (NSC) and NSC multi-functional acrylate. PETA: pentaerythritol triacrylate; 2-HEA: 2-hydroxyethyl acrylate.

In this study, a nano-silica-core multifunctional acrylate (NSC-MF acrylate; Nano Polychem, Daejeon-si, Korea) was prepared by directly replacing the surface of nano-silica with acrylate. To prepare nano-silica particles from TEOS, a sol-gel method was used. In the manufacturing process, the size of the nanoparticles is 10–200 nm. As the size of the filler material increases, an optical disorder phenomenon occurs in the visible light/UV region. Here, particles with a size of 20–30 nm were selected through pressure filtering. To modify the nano-silica, briefly, a condensation reaction was used to directly attach acrylates on the surface of prepared pure silica. Two acrylates, 2-hydroxyethyl acrylate (2HEA) and pentaerythritol triacrylate (PETA), were used in this experiment (Figure 2). The condensation reaction with the hydroxy group of 2HEA/PETA was carried out using the hydroxy group present on the surface of the refined silica. In general, one-pot method using TEOS and acrylate silane at one time is also used to make acrylate-introduced silica on the surface [33]. In this study, we have selected this method to improve the compatibility in the blending process and to control the viscosity in the silica dispersion process. A composite resin was then prepared using NCS-MF acrylate and its shrinkage measured as well as the effects of compositional variation on its properties. Similarly, Micure HP-8 was used as a photoinitiator.

The final NSC-MF acrylate was composed of 50% nano-silica, 35% PETA, and 15% 2HEA by weight. Three other acrylates, i.e., PETA, TMPTA, and bisphenol A glycidyl methacrylate (BIS-GMA), were selected to blend with the prepared filler materials under a variety of conditions to form the composite materials. As NSC-MF acrylate and each of the acrylates were mixed at weight ratios of 100:0, 80:20, 60:40, 40:60, 20:80, and 0:100, the proportions of the nano-silica were designed to be 50, 40, 30, 20, 10, and 0 wt. %, respectively.

### 2.3. Photo-Differential Scanning Calorimetry

Photo-differential scanning calorimetry (DSC) experiments were conducted using a DSC (Q-200, TA Instruments, Chicago, IL, USA) equipped with a photo-calorimetric accessory. The light source was Spot-Cure (Omnicure-s2000, Excelitas, Waltham, MA, USA). Spot-Cure uses a 200 W high-pressure mercury vapor lamp. The light intensity was determined by placing an empty open aluminum DSC pan on the sample cell. The UV light intensity that the sample was subjected to was 10, 25, 50, or 100 mW/cm^2^ over a wavelength range of 300–545 nm. The sample weight was about 3 mg. Finally, all measurements were carried out at 25 °C.

### 2.4. Shrinkage Measurements

The amount of shrinkage in each system was measured using a linometer (Plustek, Seoul, Korea). Briefly, a predefined amount of specimen was loaded onto a stainless steel plate and covered with a sliding glass. This was then placed on the displacement measurement sensor and transducer, after which the slide glass on top was fixed in place. When shrinkage of the specimen during UV irradiation occurred, the upward movement of the stainless steel plate was recorded over time. Finally, this axial shrinkage in the vertical direction was converted into volume data to estimate the volumetric shrinkage. The UV light intensity during curing was 10 mW/cm^2^ over a wavelength range of 300–545 nm, the sample volume was about 1 mL, and measurements were carried out at 25 °C.

The linometer measures only the material shrinkage in the axial direction. Generally, the volume shrinkage ratio is three times the linear shrinkage ratio according to the following equation. However, the linear shrinkage and the volume shrinkage are almost the same for a sample in which the aspect ratio of the sample is sufficiently large and the upper side is fixed [8,16,34].

(1) $$\begin{array}{l} \mathrm{Volume}\;\mathrm{of}\;\mathrm{Original}\;\mathrm{Cubic}\;(\mathrm{Edge}\;\mathrm{length}\;\mathrm{is}\;X)=X^{3}\\ \mathrm{Volume}\;\mathrm{of}\;\mathrm{Shrinkage}\;\mathrm{Cubic}\;(\mathrm{Edge}\;\mathrm{length}\;\mathrm{is}\;X-a,\;a\;\mathrm{is}\;\mathrm{linear}\;\mathrm{shrinkage}\;\mathrm{ratio})=({X-a}^{3})\\ =X^{3}-3aX^{2}+3a^{2}X-a^{3}\;(a\;\mathrm{is}\;\mathrm{small}\;,\;\mathrm{so}\;\mathrm{we}\;\mathrm{can}\;\mathrm{ignore}\;a^{2}\;\mathrm{and}\;a^{3})\\ =X^{2}\;(X-3a) \end{array}$$

## 3. Results and Discussion

**Figure 3:**
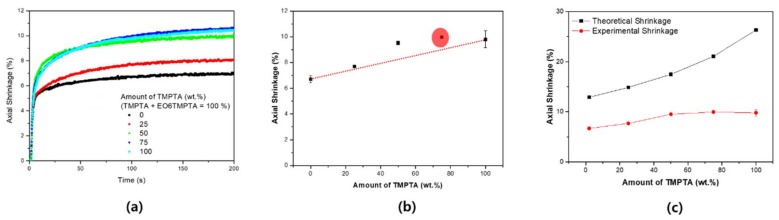
The shrinkage ratio of the binary system: (**a**) real-time axial shrinkage; (**b**) maximum shrinkage of the binary monomer system (red circle: highest shrinkage point); and (**c**) comparison of theoretical shrinkage and experimental shrinkage.

In the shrinkage evaluation of TMPTA and EO6TMPTA, the shrinkage of pure TMPTA was determined to be 9.8% and that for pure EO6TMPTA to be 6.7% (Figure 3a). Thus, the shrinkage of TMPTA was approximately 40% greater than that of EO6TMPTA.

The results show dependence of shrinkage on the ratio of the two materials in the binary system (Figure 3b). If we assume that the percentage shrinkage is determined by the mixing ratios of the two pure materials, we derive the following equation:

(2) $$Shrinkage\;ratio\;of\;binary\;system=6.7+3.1\;\times \;R_{TMPTA}$$

where *R_TMPTA_* refers to the weight fraction of TMPTA. Based on the above formula, the shrinkages calculated for 25, 50, and 75 wt. % TMPTA should be 7.48%, 8.25%, and 9.03%, respectively. However, the experimental values were 7.7%, 9.53%, and 9.98%, respectively. Not only did these values not fit the above linear proportional relationship, they also include a value that is higher than when TMPTA alone is used. The shrinkage ratio therefore was the highest at 75 wt. % TMPTA. Figure 3c shows the theoretical shrinkage rate. The theoretical shrinkage can be calculated by the following formula [35].

(3) $$\begin{array}{c} Theoretical\;Fully\;Conversion\;Shrinkage\;\left(\%\right)\\ \;\;\;\;\;\;\;\;=-1.38+2668\;\times \;\frac{Functional\;Number}{Molecular\;weight} \end{array}$$

As the content of TMPTA increases, the theoretical shrinkage increases sharply. The theoretical shrinkage is proportional to the number of functional groups and inversely proportional to the molecular weight. Therefore, in the case of TMPTA and TMPEOTA having the same functional group number, the shrinkage ratio of TMPTA having a small molecular weight is large. At 100% composition of each, the result follows this tendency. However, this tendency is different in the mixture system. Complementary experiments are needed to observe these changes.

**Figure 4:**
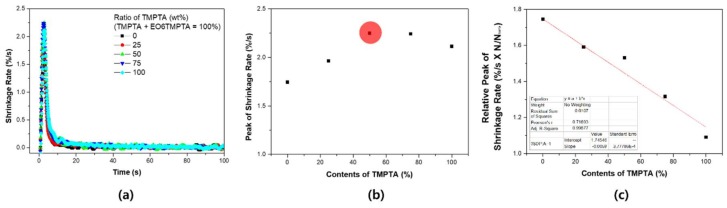
Shrinkage rate of blending system: (**a**) shrinkage rate; (**b**) maximum shrinkage rate of the binary monomer system (red circle: highest heat flow point); and (**c**) relative shrinkage rate with acrylate concentration.

Figure 4 shows the contraction rate (%/s) calculated based on shrinkage evaluation. Figure 4b shows the maximum shrinkage rate, and the velocity is not proportional to the ratio of the two materials. However, the rate of shrinkage according to the ratio of the functional group of acrylate shows a completely different tendency (Figure 4c). As the content of TMPTA increases, the maximum shrinkage rate decreases linearly. The decrease tendency is very significant at the R^2^ = 0.998 level. The acrylate population of TMPTA (Mw: 289) is more than 1.93 times that of EO6TMPTA (Mw: 560). Therefore, the reaction rate of TMPTA should be measured higher if it has the same reactivity. The absolute reaction rate is high for TMPTA, but EO6TMPTA is relatively large for relative ratio. In addition, it shows the feature that the heat flow generated is increased while mixing the materials.

**Figure 5:**
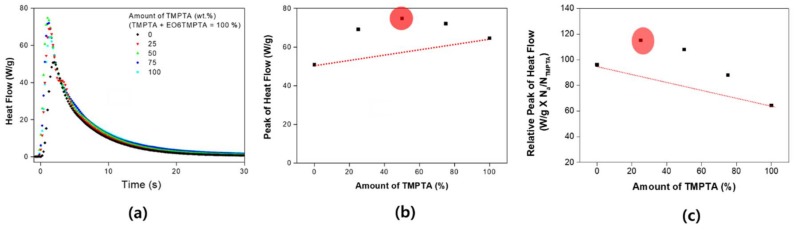
Heat flow of blending system: (**a**) real-time heat flow; (**b**) maximum heat flow of the binary monomer system (red circle: highest heat flow point); and (**c**) comparison of the relative value based on acrylate ratio.

The observed non-linearity of shrinkage was further analyzed using photo-DSC. Changes in the reaction rate were observed by changing the UV intensity. Generally, as the UV intensity increases, the reaction rate also increases. However, as can be seen in Figure 5, the curing rate did not change linearly either. Observing the results for the maximum heat flow in Figure 5, the fastest curing rate was obtained for a 50% mixing ratio. When comparing the maximum rate of reaction by peak of heat flow, the maximum value is determined at a certain rate without increasing linearly with the ratio of the two materials. In addition, it can be seen that the reaction rate proportional to the number of acrylates shows a completely different tendency. Generally, if the density of the acrylate is high, the reaction rate and the conversion rate are expected to be high. However, it can be assumed that the monomer is short in length and low in flexibility for high density. In the environment with high density of functional groups, the cage effect is likely to be expressed during the reaction. This is because the flexibility of the reacted material decreases sharply. Therefore, in the process of increasing the density of the functional groups, the factors that increase the reaction rate and the factors that inhibit the reaction increase together, so that the complex factors affect the UV curing [36].

**Figure 6:**
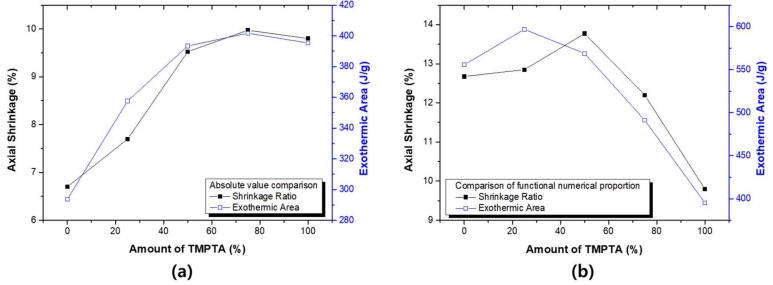
Comparative evaluation of the shrinkage ratio and the exothermic area: (**a**) comparison of the absolute value; and (**b**) comparison of the relative value based on acrylate ratio.

The heat flow obtained from the Photo-DSC shows the instantaneous calorific value of the material. Therefore, the exothermic value (or area) in the reaction process can be obtained by integrating the results. Since acrylates have a constant heat of reaction, conversion ratio can be deduced through exothermic values. Absolute value comparisons can also be used to approximate the acrylate population involved in the reaction [17,37,38]. Figure 6 compares the results of the previous shrinkage test with the exothermic area of photo-DSC. It can be seen that the trends of the two test results are almost identical in the absolute value comparison. The shrinkage phenomenon is a result proportional to the reaction ratio of the material. Therefore, the higher the reaction ratio of the material, the higher the shrinkage ratio. Based on the results, the shrinkage test and the photo-DSC evaluation are complementary methods. The correction value according to the acrylate ratio shows a slightly different tendency. Shrinkage and exothermic areas change in proportion to the ratio of the two materials, but there is a difference in the ratio with the maximum value. The shrinkage ratio is the result of measuring changes in the bulk material, and at the same time, the flexibility and conformation of the material are all reflected.

**Figure 7:**
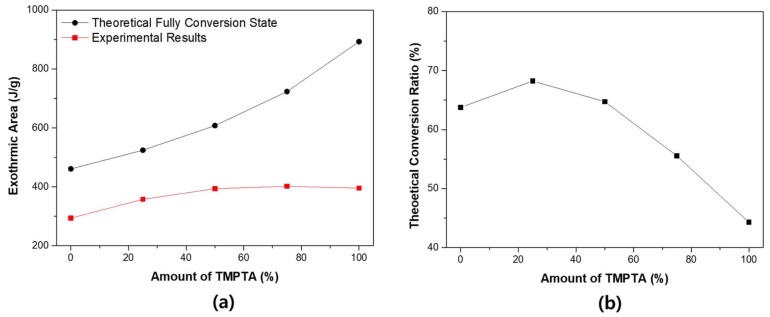
The theoretical double bond conversion ratio of the binary monomer system: (**a**) theoretical/experimental total calorific value (exothermic area); and (**b**) theoretical conversion ratio.

Photo-DSC can be used to measure the theoretical conversion ratio. When TMTPA and TMPEOTA are mixed, the population of acrylic functional group in the system are changed. Figure 7 shows the theoretical conversion ratio of photo-DSC. Figure 7a shows the theoretical calorific value at 100% conversion state of each system and the experimental measurement results of each system.

(4) $$\begin{array}{l} Theoretical\;Conversion\;Ratio\;\left(\%\right)\\ =Exothermic\;Area\;\left(J/g\right)/Theoretical\;Fully\;Conversion\;State\;\left(J/g\right)\; \end{array}$$

The reaction sequence of the theoretical acrylate functional group is ΔH_theory_ = 86 kJ/mol [39]. Based on this, the theoretical total calorific value of each system can be calculated. As the TMPTA content increases, the actual total calorific value increases. However, the increase in theoretical total calorific value is greater. As a result, the theoretical conversion ratio tends to decrease when the content of TMPTA increases. This result can be understood as the phenomenon that the cage effect of TMPTA. As TMPTA increases, the reaction rate increases and the unreacted material also increases.

**Figure 8:**
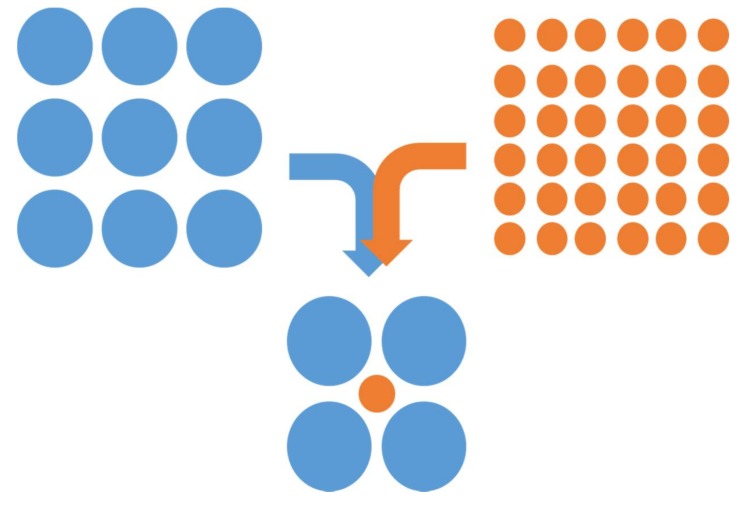
Schematic of the nano-porous effect formation for the binary monomer system with two monomers of different size.

Based on molecular weight, EO6TMPTA has a relatively higher shrinkage rate than TMPTA. This tendency is due to the excellent mobility of EO6TMPTA. Mobility of EO6TMPTA is increased by ethoylated group. These ethoylated groups serve to expand the free space of EO6TMPTA [8]. This difference in shrinkage and reaction rate behavior may be due to a nano-porous effect. As shown schematically in Figure 8, the reactivity is maximized when small molecules are located between large molecules. In small structures made of such blends of differently sized molecules, the input of energy is extremely important, significantly affecting the liquidity and density of acrylate.

**Figure 9:**
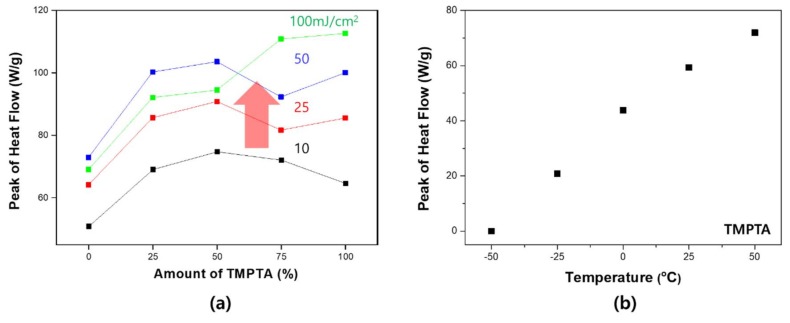
Heat flow of the binary monomer system in accordance with the UV intensity: (**a**) by UV intensity; and (**b**) by temperature.

To investigate these effects, the curing behavior was investigated by varying the UV intensity. As shown in Figure 9, the curve changed as the UV intensity increased. Specifically, the reactivity of the TMPTA was confirmed to have increased sharply. The peaks for 100 wt. % TMPTA increased dramatically in proportion to the UV intensity, whereas EO6TMPTA (=TMPTA 0 wt. %) did not change significantly. Comparing TMPTA with EO6TMPTA, the molecular mobility of TMPTA is relatively low, since TMPTA has a higher *T_g_*. As the reaction progresses, a portion of the TMPTA that is curing undergoes a sharp decrease in mobility. Eventually, only acrylate sites that do not participate in the reaction remain. Therefore, when EO6TMPTA is mixed with TMPTA, the latter’s unreacted sites can participate in the reaction continuously. In addition, as the intensity increases and the internal energy increases, the mobility of the molecules relatively increases, improving their reactivity. For this reason, there is a difference between the two materials in the amount of change of the peak reactivity upon increasing the UV intensity. These results are more apparent when the temperature-dependent photo-DSC results are observed. The *T_g_* of TMPTA is about 62 °C. When the curing progresses partly to form a three-dimensional network, the *T_g_* of the polymer corresponding to the portion continuously increases to reach 62 °C. Therefore, the farther the temperature of the peripheral part is from the *T_g_*, the more rapidly the mobility decreases. The maximum response increases linearly with increasing ambient temperature from −50 to 50 °C. As the temperature increases, the flow rate of the material changes. Therefore, the reaction rate may increase. However, according to the results of Park et al. The material having a low specific *T_g_* of the material tends not to change with temperature have [10]. As a result, Figure 9 shows that the difference in reactivity due to mobility can affect the mixed system of materials.

**Figure 10:**
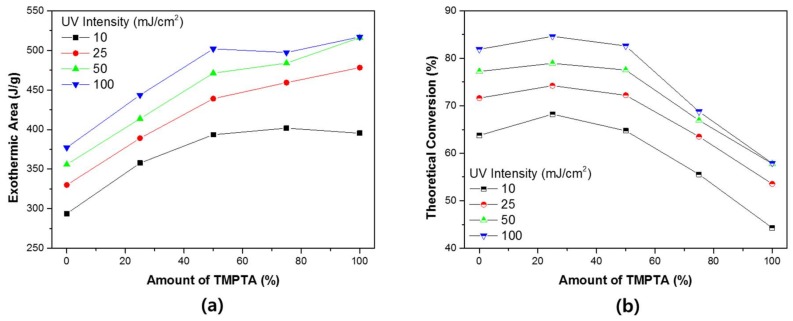
The theoretical double bond conversion ratio of the binary monomer system in accordance with the UV intensity: (**a**) experimental results; and (**b**) theoretical conversion.

As the UV conditions change, not only the reaction rate of each system changes, but also the conversion ratio of the whole system changes. Figure 10 shows the conversion ratio calculated based on the total calorific value and the theoretical calorific value of each system. As the UV intensity increases, the overall calorific value also increases. The conversion ratio based on this trend tends to decrease as TMPTA increases. The overall tendency does not change with changes in UV intensity. However, as TMPTA increases, the relative increase in conversion ratio is large. When the nano-porous effect is expressed in the system, the cure rate increases but does not increase to the conversion ratio. This can be understood as a phenomenon depending on the density of acrylate. This is because the density of the acrylate influences the formation of the network structure in the three-dimensional space [40,41].

**Figure 11:**
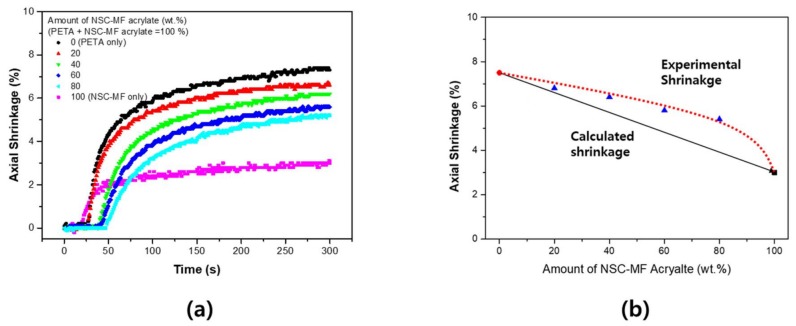
Shrinkage of the blended system of nano-silica-core multi-functional acrylate (NSC-MF acrylate) with PETA: (**a**) shrinkage over time depending on relative NSC-MF acrylate content; and (**b**) maximum shrinkage depending on relative NSC-MF acrylate content.

Figure 11 presents the results of the evaluation of the system with PETA blended with NSC-MF acrylate. Since mainly PETA was used to treat the surface of the nano-silica filler, blending between the two materials was excellent. As mentioned above, the nano-porous effect occurs if two materials of different sizes are mixed. Since NSC-MF acrylate has a very large molecular mass, PETA could fill the space between its molecules. Mixing PETA with NSC-MF is similar to increasing the proportion of silica not participating in the reaction with PETA. In addition, since the –OH groups of PETA interact with the surfaces of silica particles, the nano-porous effect cannot be expected. Therefore, as the content of NSC-MF increases, the shrinkage rate decreased linearly.

**Figure 12:**
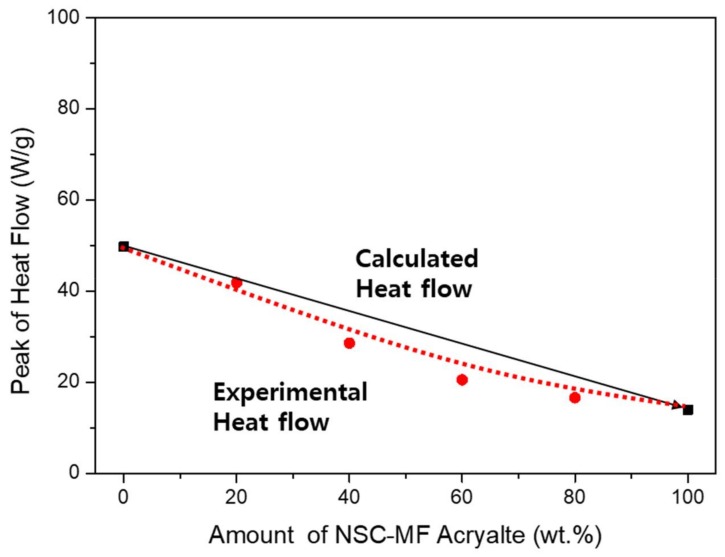
Peak of heat flow for the blended system of NSC-MF acrylate and PETA.

Figure 12 shows the photo-DSC results. First, the system with PETA revealed a mostly linear dependence, similar to the shrinkage tests. There were no significant differences in the blending characteristics compared to calculated values, because the PETA structure is similar to the surface-substituted structure of NSC-MF acrylate. Finally, no phase separation occurred between PETA and NSC-MF acrylate.

**Figure 13:**
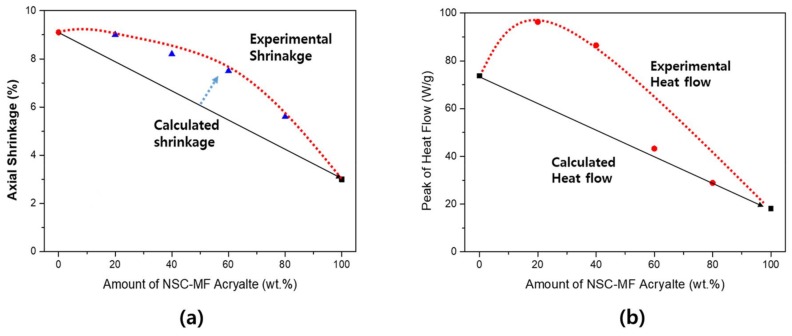
Blended system of NSC-MF acrylate and TMPTA: (**a**) shrinkage ratio; and (**b**) peak of heat flow.

Figure 13 shows that the variation of shrinkage is even greater in the blended system with TMPTA instead of PETA. It was found that the more the curing conditions improved, the higher was the shrinkage of TMPTA. The shrinkage relatively increased (compared to calculated shrinkage) in the mixed conditions because NSC-MF acrylate enhanced the mobility of TMPTA. TMPTA with relatively free movement was dispersed among the large NSC-MF macromers, improving its reactivity. Therefore, unlike PETA, the shrinkage rate did not decrease linearly. The maintained shrinkage ratio despite the increase in NSC-MF acrylate content can be explained by the aforementioned nano-porous effect. Photo-DSC results for the system with TMPTA; the curing rate relatively increased under blended conditions. The nano-porous effect may have increased with molecular size. Since TMPTA has a high *T_g_* and self-inhibiting happens during the reaction, it is important to ensure sufficient molecular mobility. Thus, the introduction of the macromolecular NSC-MF acrylate forms a structure in which the mobility is constantly ensured, maximizing the reactivity. Similar tendencies are observed between the shrinkage measurement and the photo-DSC, but there is a difference between the two methods that similar to the binary monomer system. Unlike the previous evaluation, it is difficult to predict the exact molecular weight. Compared with absolute values, this system shows a larger difference because the bulk material such as NSC-MF affects both reaction and mobility.

**Figure 14:**
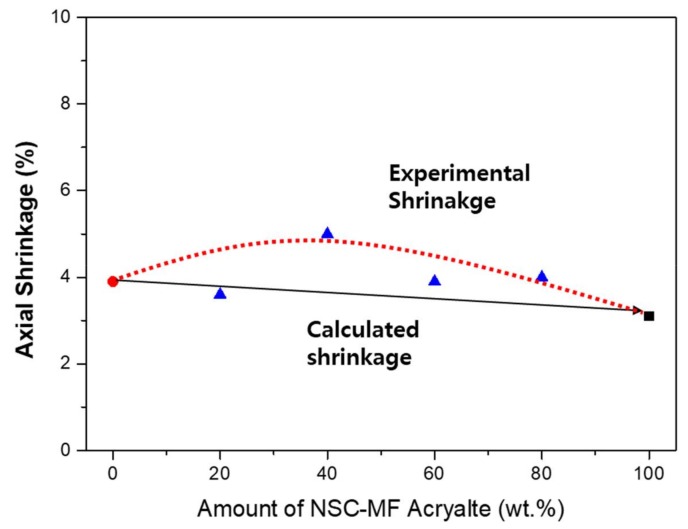
Shrinkage of the blended system of NSC-MF acrylate and BIS-GMA.

**Figure 15:**
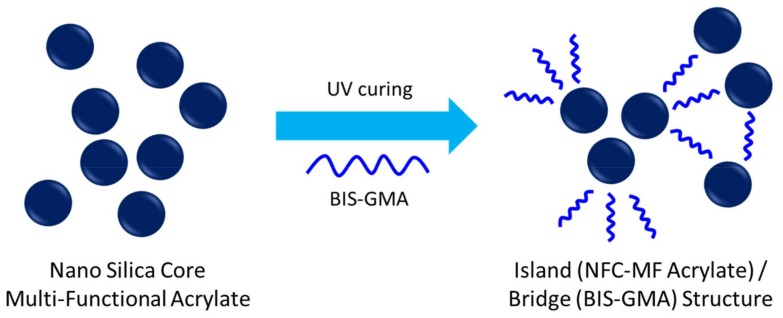
Island/bridge structure of the mixture system of NFC MF acrylate and BIS-GMA.

The same nano-porous effect between monomers and macromers was very large in the blended system with BIS-GMA (Figure 14), because island-bridge structures (schematically represented in Figure 15) could be formed due to the structural difference between BIS-GMA and NSC-MF acrylate; NSC-MF acrylate has a very large size with very low overall mobility, while BIS-GMA is a di-functional monomer with relatively low viscosity. The latter could improve the flexibility and consequently induce reactions between the functional groups.

In addition, as shown in Figure 14, the molecular chains themselves may have acted as springs to boost the overall shrinkage as the reaction continued. The change in curing properties were significant depending on the structural characteristics of the material that was blended with NSC-MF acrylate; the nano-porous effect varies greatly depending not only on the molecular size, but also on the mobilities and shapes of the molecules. There is no difference in molecular size between TMPTA and BIS-GMA, but a large difference in molecular shape. BIS-GMA with its linear structure has a greater influence on the binding between large molecules.

**Figure 16:**
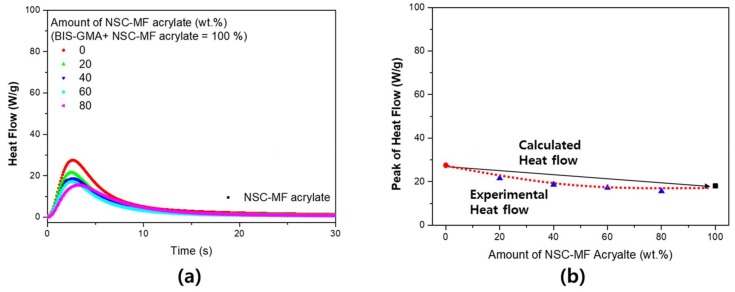
Heat flow of the NSC-MF acrylate and BIS-GMA blended system: (**a**) real-time heat flow; and (**b**) maximum heat flow of the blended system.

In the system with BIS-GMA, on the other hand, the reaction rate did not increase significantly. As shown in Figure 16, the reaction rate appeared to be mostly constant without a dependence on the blending conditions, whereas the previous assessment for shrinkage revealed a significant island-bridge structure effect. Since BIS-GMA has a di-functional structure, the reactivity is relatively high compared to PETA and TMPTA. Therefore, an increase in reactivity due to structural changes should not be expected. Thus, while the rate of shrinkage increased, the rate of reaction did not change.

## 4. Conclusions

The ultraviolet curing characteristics of blended binary and composite systems are investigated. The shrinkage in these systems is not proportional to the shrinkage of the pure molecules and the weight ratios between the individual molecules. When two materials of different sizes are mixed, a relative increase in reactivity and shrinkage with partial mobility improvement is confirmed. The changes in the reaction rates are attributed to varying shapes and sizes of the molecules. The exact porous structure, which varies with the molecule size, is determined to be a factor the reactivity depended on. In addition, in the composite system using a nano-silica-core multifunctional acrylate filler, there is a difference in reactivity depending on the exact blending conditions of the materials, with results similar to those of the binary system. However, when an oligomer acrylate is used instead of a linear monomer acrylate, the reaction rate relatively increases while the shrinkage tendency decreases. The latter is assumed to be due to formed island/bridge structures.
